# EVALUATION OF PROGRAMS AND SERVICES FOR CAREGIVERS OF PEOPLE LIVING WITH DEMENTIA

**DOI:** 10.1093/geroni/igae098.1898

**Published:** 2024-12-31

**Authors:** Lauren Stratton, Sam Fazio

**Affiliations:** Chair; Discussant

## Abstract

The majority of care provided to people living with dementia is from family or informal caregivers. There is currently a lack of evidence-based programs and services that are available online or virtually to informal caregivers. This symposium consists of three studies, including the evaluation of two programs and the 24/7 Helpline that are available to informal caregivers of people living with dementia through the Alzheimer’s Association. The novel findings presented in this symposium will focus on the full impact these programs and services have on caregivers and address the gap in evidence-based programming. First, Judge will discuss the evaluation of a new program, The Empowered Caregiver, which highlights positive efficacy and acceptability data. Second, Grant will present the results of an evaluation for both an online and virtual program, Managing Money: A Caregiver’s Guide to Finances. Lastly, Hodgson will discuss three iterative studies that evaluated the Alzheimer’s Association 24/7 Helpline, which provides 24/7 support over the phone, including care consultations. Fazio will serve as the discussant to draw attention to current gaps in caregiving intervention literature, the importance of evidence-based programs and services, and the future direction of these programs. Together, these studies contribute to our understanding of effective and accessible programs and services for caregivers.

